# A Meta-Analysis of Typhoid Diagnostic Accuracy Studies: A Recommendation to Adopt a Standardized Composite Reference

**DOI:** 10.1371/journal.pone.0142364

**Published:** 2015-11-13

**Authors:** Helen L. Storey, Ying Huang, Chris Crudder, Allison Golden, Tala de los Santos, Kenneth Hawkins

**Affiliations:** 1 Diagnostics Program, PATH, Seattle, Washington, United States of America; 2 Fred Hutchinson Cancer Research Center, Seattle, Washington, United States of America; Royal Tropical Institute, NETHERLANDS

## Abstract

Novel typhoid diagnostics currently under development have the potential to improve clinical care, surveillance, and the disease burden estimates that support vaccine introduction. Blood culture is most often used as the reference method to evaluate the accuracy of new typhoid tests; however, it is recognized to be an imperfect gold standard. If no single gold standard test exists, use of a composite reference standard (CRS) can improve estimation of diagnostic accuracy. Numerous studies have used a CRS to evaluate new typhoid diagnostics; however, there is no consensus on an appropriate CRS. In order to evaluate existing tests for use as a reference test or inclusion in a CRS, we performed a systematic review of the typhoid literature to include all index/reference test combinations observed. We described the landscape of comparisons performed, showed results of a meta-analysis on the accuracy of the more common combinations, and evaluated sources of variability based on study quality. This wide-ranging meta-analysis suggests that no single test has sufficiently good performance but some existing diagnostics may be useful as part of a CRS. Additionally, based on findings from the meta-analysis and a constructed numerical example demonstrating the use of CRS, we proposed necessary criteria and potential components of a typhoid CRS to guide future recommendations. Agreement and adoption by all investigators of a standardized CRS is requisite, and would improve comparison of new diagnostics across independent studies, leading to the identification of a better reference test and improved confidence in prevalence estimates.

## Introduction

Typhoid fever causes considerable disease burden, with recent estimates at 21.6 million illnesses in 2000 and 26.9 million in 2010 [1,2]. These estimates are extrapolated from limited population-based studies and further compromised by the poor accuracy of current typhoid diagnostics. The most accepted method used for typhoid detection is blood culture [3]. It is desirable to diagnose typhoid fever because of its perfect specificity, but with sensitivity around 50% in most clinical settings, there is much room for improvement [4,5]. New diagnostic tests for typhoid fever are in development which may relieve this shortfall; however, a problem remains in regard to determining the best reference test with which to evaluate new diagnostics [6,7]. Using a reference test with imperfect diagnostic accuracy may cause newer technologies to appear better or worse than they really are, which in turn clouds the evaluation of their utility as a tool to improve disease burden estimates [8]. Additionally, to compare across index tests with statistical rigor, a common reference test should be used.

Lack of a perfect gold standard in diagnostic research is not an uncommon situation, and yet there is no universally accepted solution to the problem [9]. One method to improve diagnostic accuracy when no perfect reference test exists is to develop a composite reference standard (CRS) [10]. A CRS combines more than one imperfect diagnostic test with the goal of increasing diagnostic accuracy (compared to truth: the true presence of infection). If the individual tests in the CRS are highly specific, combining them by declaring CRS positive if either test is positive should give greater sensitivity than either test alone [10]. This would also enable combining multiple types of testing, such as direct detection of the bacteria with culture and immune detection with an antibody-based assay. Compared to other methods such as discrepant resolution and latent class analysis, an ideal consensus CRS has the advantage that it is more clearly defined, independent of the results of the index test, and more straightforward to interpret [10]. Particularly in the context of typhoid diagnostic field evaluations, the consensus CRS approach to addressing imperfect reference tests may be the most feasible and meaningful for the researchers performing the studies.

In order to evaluate tests for use as a reference test or inclusion in a CRS, we conducted a systematic review of the typhoid literature. We described the types of reference tests that are being used to evaluate new typhoid diagnostics, and summarized by meta-analysis the diagnostic accuracy of available index tests when compared to the most common reference test (blood culture), including the evaluation of variability due to study quality. We then discussed how a standardized CRS, rather than a single reference test, may improve the evaluation of new typhoid diagnostics using a constructed numerical example. Finally, based on the results of our systematic review, meta-analysis, and the constructed numerical example, we proposed recommended criteria and potential components of a CRS for consensus-building and discussion. Agreement and adoption of a standardized composite reference method would enable comparison of diagnostic performance data across independent studies, leading to improved confidence in prevalence estimates.

## Methods

### Search strategy and inclusion criteria

**Fig 1:**
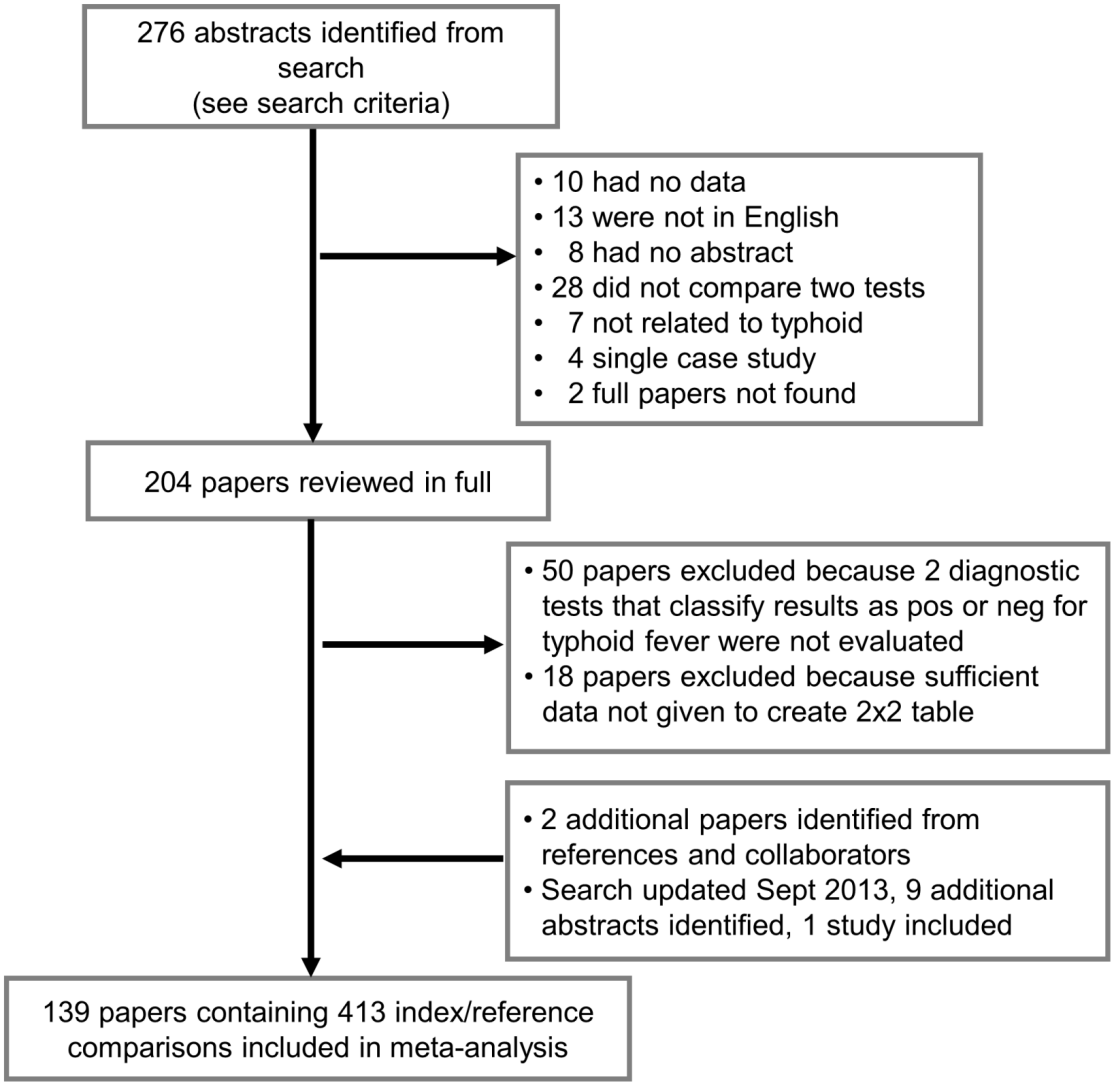
PRISMA flowchart. Study flow depicting search strategy, inclusion/exclusion criteria, and summary of systematic review.

We performed a review and meta-analysis using the PRISMA reporting guidelines (S1 Table) [11]. In May 2013, a detailed search strategy was designed to identify all papers in English that evaluated diagnostic tests for the detection of typhoid fever among humans. If papers considered typhoid fever to include *Salmonella typhi* and *S*. *paratyphi A*, the data were included as such in our review. The following databases were included: Pubmed, EMBASE, and ISI Web of Science (S2 Table). In all, 276 papers were identified from our searches (Fig 1). After reviewing the abstracts, 72 papers were excluded based on the following: had no original data (10), were not in English (13), had no abstract available (8), did not compare two diagnostic tests (28), were not related to typhoid (7), described single case studies (4), and no full paper was found (2). The remaining papers were reviewed in full (204), and further exclusions were made if two diagnostic tests were not evaluated that dichotomized result as positive or negative for typhoid fever (50), or sufficient data were not given to confidently infer all values of the contingency table (18). A diagnosis based on clinical indicators was considered a diagnostic test if classified as positive or negative. Additional papers were identified from references or collaborators (2). The search strategy was updated September 2013 and nine additional abstracts were identified, of which one paper was added to the study. In total, 413 index/reference comparisons from 139 papers were included in the meta-analysis (Fig 1) [12–181].

### Evaluation of studies

Data were extracted from the selected studies with consensus from two investigators and using Microsoft Excel and Access. Many of the included papers reported results for more than one comparison of index and reference tests (S3 Table). Each comparison was classified by the method of detection of the index and reference tests, and broadly grouped in the following six categories: clinical indicators, antibody, antigen, nucleic acid, viable bacteria, or composite. Each index and reference test was specified in as much detail as was given, including specimen type, sample size, and commercial availability (manufacturer and test kit specified). Also recorded were the number of samples analyzed, and the number of true positives, false positives, true negatives, and false negatives for each comparison. If the absolute value for each cell of the 2x2 contingency table was not reported in the paper, it was calculated from reported sensitivities and specificities where possible. Some cohort studies included additional healthy controls as part of their analysis, and that data was excluded from this analysis. If a case-control study analyzed data separately for different control groups, all control groups were combined in this analysis. Where possible, the same antibody test separately evaluating more than one Ig isotype were combined. The data was included separately when not possible to combine.

Each study was evaluated using the QUADAS-2 tool, which assesses the quality of diagnostic accuracy studies [182]. The QUADAS-2 tool includes four domains: patient selection, index test, reference standard, and flow and timing. All domains are evaluated for risk of bias, and the first three domains are also evaluated for concerns regarding applicability (Table 1). Measures of bias or applicability were assessed as potential covariates to adjust for in the meta-regression analysis.

**Table 1:** Quality assessment of diagnostic accuracy studies.

Domain	Criteria	Conclusion	Covariate^2^
Patient selection	A consecutive or random sample of patients was enrolled	If no, then *risk of bias* is high	High compared to low
	A case-control design was avoided	If no, then *risk of bias* is high	High compared to low
	The study avoided inappropriate exclusions	If no, then *risk of bias* is high	High compared to low
	The included patients were individuals suspected of having typhoid fever and the diagnostics were used to diagnose the patients	If no, then *concern of applicability* is high	
Index test	A threshold for the test result was pre-specified	If no, then *risk of bias* is high	High compared to low
	The test result was interpreted without knowledge of the reference test	If no, then *risk of bias* is high	High compared to low
	The index test aimed to diagnose acute typhoid fever	If no, then *concern of applicability* is high	
Reference test	The reference standard was likely to correctly classify the target condition of acute typhoid fever^1^ (Recorded as “unclear” if the reference test was not a more commonly used test, the methods were not well described, and it was unclear if the results were interpreted without knowledge of the index test)	If no, then *risk of bias* is high	High compared to low
	The reference test was interpreted without knowledge of the index test	If no, then *risk of bias* is high	High compared to low
	The reference standard aimed to diagnose acute typhoid fever	If no, then *concern of applicability* is high	
Flow and timing	There was an appropriate interval between index test and reference test	If no, then *risk of bias* is high	High compared to low
	All patients received a reference standard	If no, then *risk of bias* is high	High compared to low
	All patients received the same reference standard	If no, then *risk of bias* is high	High compared to low
	All patients included in the analysis	If no, then *risk of bias* is high	High compared to low

Summary of variables included in the QUADAS-2 tool assessing the quality of diagnostic accuracy studies. The criteria determined a study’s risk of bias or concern of applicability. When the domain-specific criteria were not met, the study had a high risk of bias or concern of applicability with respect to that domain. When the domain-specific criteria were all unclear, the risk of bias or concern of applicability was unclear.

^1^ The currently available tests to detect typhoid fever are not sufficiently accurate; therefore, this question was problematic.

^2^ “Unclear” = missing.

### Analysis and statistical methods

**Fig 2:**
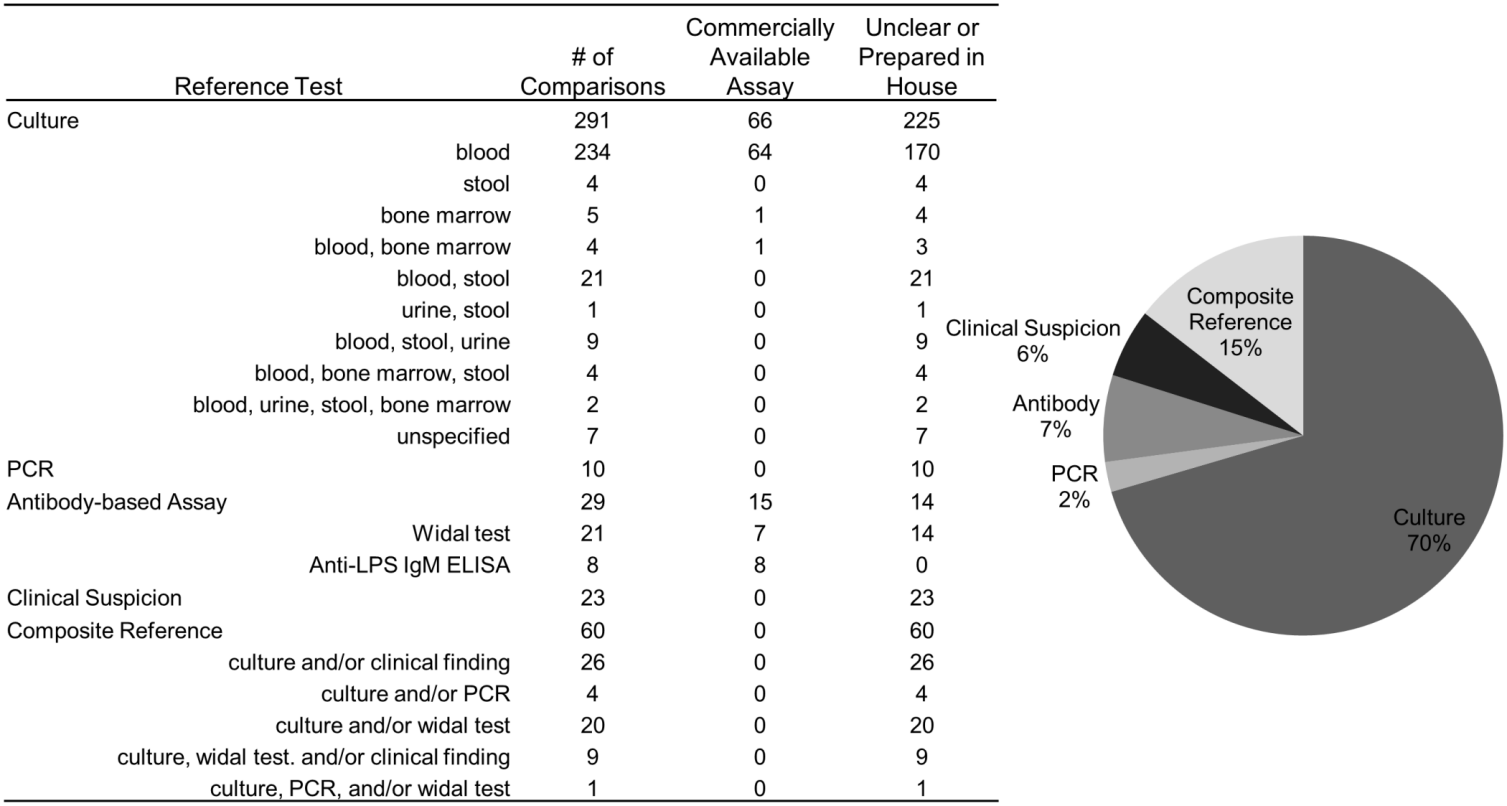
Comparisons by reference test. Summary of the 139 papers by reference test, including 413 index/reference comparisons. Of the culture reference tests, 80% were blood culture, making up 57% of all reference tests.

All index/reference combinations were first summarized by type of reference standard used (Fig 2). Index tests were then categorized into five broad groups, based on the detection of nucleic acid, antigen, antibody, viable bacteria, or clinical features (including composite tests). Diagnostic accuracies of individual index tests were analyzed using meta-analysis when particular index/reference combinations were present in more than five comparisons, to increase the relevance of the conclusions. The majority of meta-analyses were based on combinations using blood culture as the reference test. Statistical analyses were performed using STATA^®^ 11.2 (StataCorp, TX, USA) and R version 3.1.3 (https://www.r-project.org/). Summary results were calculated in the meta-analysis using bivariate random effects binomial regression (STATA command: *metandi*), and listed in the text as follows: summary sensitivity (Sens) (95% Confidence Interval, CI), summary specificity (Spec) (95% CI), and summary diagnostic odds ratio (DOR) (95% CI) [183,184]. Sources of heterogeneity in the summary estimates were investigated in the meta-regression analysis, also using bivariate random effects binomial regression (STATA command: *midas*) [185]. Potential covariates were transformed into dichotomous variables and evaluated as appropriate for each index test (Table 1) [182]. The hierarchical summary receiver operating characteristic curves were also generated [186].

### Constructed numerical example

To illustrate how the observed sensitivity and specificity of an index test may vary compared to individual reference tests or a CRS, a constructed numerical example was generated with assumptions on disease prevalence and the joint distribution of index and reference tests conditional on disease status. The assessed CRS was fever positive *and* either test A positive *or* test B positive, i.e. CRS = ((fever) AND (test A positive) OR (test B positive)). Because test accuracy compared to truth (true presence of infection) is not measurable, and no current reference test is an adequate proxy for truth, assumptions were made regarding the prevalence of disease, as well as true test characteristics of the index test, test A, and test B.

Prevalence of typhoid fever was assumed to be 20%, though many factors vary prevalence estimates, such as participant ages, region, and surveillance method. Studies conducting active community-based surveillance have determined the prevalence of typhoid fever among blood cultures taken to be 2.3%, 2.8%, and 5.0% in five Asian countries, Kenya, and Bangladesh, respectively [187–189]. However, other non-surveillance hospital-based studies have observed the proportion of suspected typhoid patients with positive blood cultures to be 72.8% and 30.4% in Vietnam and sub-Saharan Africa, respectively [86,190]. A prevalence of 20% was selected for illustration purposes. Fever, defined by the World Health Organization (WHO) as ≥37.5°C for ≥3 days, compared to truth was 80% sensitive and 20% specific. Most typhoid fever patients have fever at clinical presentation, but there are many other causes of fever [191]. Additionally, in a recent study, the WHO case definition for suspected typhoid fever demonstrated sensitivity and specificity of 82.6% and 36.3%, respectively [3,169]. Fever is assumed to be independent of all diagnostic tests, conditional on disease. Test A was considered to be blood culture because of its wide appeal, and the sensitivity and specificity of blood culture compared to truth was 50% and 100%, respectively [4,5]. Test B was a hypothetical second test, which was assumed to be 85% sensitive and specific. Test A and test B were independent, conditional on disease. The index test compared to truth was assumed to be 80% sensitive and 90% specific, as the accuracy of a new diagnostic test would probably need to reach these standards [192].

The CRS based on individual tests described above had 74% sensitivity and 88% specificity compared to truth (S1 Text). Two situations were evaluated: (1) independence between index test, test A, and test B, conditional on disease; and (2) dependence between either index test and test A, or index test and test B, conditional on disease. We constructed these situations to demonstrate how a range of conditional dependence may vary the results; however, it is unlikely that technologically the index test would be unrelated to either test A or test B due to related biomarkers or methods for detecting biomarkers. Though the degree of correlation is largely unknown, the minimum and maximum values it can achieve can be determined by the accuracy of the individual tests [193]. Assuming various conditional dependence structures between tests, the following sensitivities and specificities were calculated based on Bayes’ theorem: (1) index test compared to test A; (2) index test compared to test B; and (3) index test compared to CRS (S1 Text). Example R code for computing these values is included in S1 Text as well.

## Results

### Systematic review

Among the 413 index/reference combinations identified, numerous reference tests were used to evaluate typhoid diagnostic assays. The largest category of reference tests was culture based, accounting for 291 of 413 comparisons, or 70% of all reference tests (Fig 2). Of these, the majority of culture-based reference tests (234) used blood specimens for culture; however, specimen volume, length of culture time, culture media, and sub-culturing techniques varied across studies. Other reference test categories and their corresponding number of comparisons included polymerase chain reaction (PCR)-based assays (10), antibody-based assays (29), clinical suspicion (23), and composite references (60). The composite references identified were variable and many included some clinical assessment along with a laboratory-based diagnostic assay. Only 20% (81/413) of all reference tests were commercially available assays, while other comparisons used reference tests that were developed in house or did not specify how the assay was acquired. Four comparisons were dropped from the meta-analysis because no positives were detected by the reference test (true positives + false negatives = 0). Of the remaining comparisons, 286 had a high risk of bias and 22 had a high concern of applicability in any domain. Other participant characteristics that were initially examined as sources of heterogeneity were later not used in the analysis due to a large proportion of comparisons missing data (176 missing age, 189 missing fever duration, and 294 missing prior antibiotic use).

There were 60 comparisons from 20 studies that evaluated an index test for typhoid detection compared to a CRS. The CRS included the following: culture, PCR, and Widal assay (1); culture and PCR (1); culture and Widal assay (10); and culture and clinical features (9). Exact criteria for a positive reference test result varied from study to study, such as Widal assay titer cutoff, culture specimen and methods, PCR technique, and specifics of clinical features (Fig 2). Reference tests may be combined in a CRS definition by either an “and” or “or” statement. Most of the comparisons evaluated used an “or” statement to combine reference tests (both test A and test B are measured, and a positive test result by either A ***or*** B classifies the person as CRS positive).

### Meta-analysis

#### Index tests detecting viable bacteria

From the systematic review, 18 comparisons detected viable bacteria by culture as the index test. This group of assays had considerable variability in methods of culturing, with few studies using automated culture systems (2). The year of publication ranged from 1979 to 2013. The specimens cultured included variable quantities of blood (13), blood clot (1), bone marrow (1), urine (2), and stool (1). Reference tests included cultures using a different specimen (7), such as blood culture compared to bone marrow culture, as well as PCR assays (2), clinical features (6), and composite references combining more than one reference test (3). The only index and reference test combination with at least five comparisons was blood culture compared to bone marrow culture, which gave the following summary results: Sens = 68% (52–81%), Spec = 75% (35–94%), and DOR = 6 (2–27).

#### Index tests detecting *S*. *typhi* antigen

The 66 assays that detected antigen included an assortment of analyte specificity, specimen type, and reference test. The year of publication ranged from 1980 to 2013. The specimens assayed included serum (34), blood culture supernatant or broth (22), and urine (10). The techniques used for antigen detection varied and all but two were in-house preparations. Reference tests included various types of culture (43), antibody-based assays (8), PCR (1), and composite references (14). Of the 66 comparisons, there were no combinations of index and reference tests with five or more comparisons.

#### Index tests using clinical features or more than one assay

There were three comparisons with index tests that included clinical features and an antibody-based assay, one comparison with only clinical features, one comparison with more than one antibody-based assay, and three comparisons that combined blood culture and an antibody-based assay. These eight comparisons varied in index test attributes as well as reference test attributes. All reference tests included blood culture and some included other reference attributes, such as clinical features (4) and culture of other specimens (1). No combinations of index and reference tests had five or more comparisons.

#### Index tests detecting *S*. *typhi* nucleic acid

Among the 23 comparisons with PCR assay as the index test, 6 were un-nested, 16 were nested, and 1 was a real-time PCR assay. The year of publication ranged from 1993 to 2012. Some of the specimens used were urine (3), stool (1), cultured blood material (2), buffy coat fraction of blood samples (1), and blood (16). Reference tests were most commonly blood culture (16), though some comparisons used another PCR assay (1), clinical features (5), or a composite reference (1). Among the 16 comparisons that evaluated a PCR-based index test to blood culture, the summary results were: Sens = 96% (88–99%), Spec = 87% (69–96%), and DOR = 191 (48–754). (Table 2)

**Table 2:** Meta-analysis results by study quality.

Index test	# of comparisons (studies)	Sensitivity (95% CI)		p-value	Specificity (95% CI)		p-value	Diagnostic odds ratio (95% CI)		Any high concern of applicability (%)
**PCR**	**16 (16)**	**0.96**	**(0.88, 0.99)**		**0.87**	**(0.69, 0.96)**		**191**	**(48, 754)**	**0.06**
*Patient selection bias*: Yes	7	0.93	(0.83, 1.00)	0.69	0.95	(0.88, 1.00)	0.04			
No	9	0.98	(0.95, 1.00)		0.76	(0.52, 1.00)				
*Index test bias*: Yes	1	0.4	(-0.37, 1.00)	0.02	0.93	(0.67, 1.00)	0.12			
No	11	0.96	(0.91, 1.00)		0.92	(0.82, 1.00)				
*Reference test bias*: Yes	1	0.99	(0.95, 1.00)	0.001	0.59	(-0.37, 1.00)	0.71			
No	11	0.97	(0.92, 1.00)		0.82	(0.64, 1.00)				
*Patient flow bias*: Yes	2	0.83	(0.43, 1.00)	0.49	0.94	(0.75, 1.00)	0.11			
No	14	0.97	(0.94, 1.00)		0.86	(0.72, 1.00)				
**Anti-LPS assay**	**33 (20)**	**0.84**	**(0.78, 0.89)**		**0.89**	**(0.83, 0.93)**		**42**	**(23, 75)**	**0.06**
*Patient selection bias*: Yes	16	0.91	(0.87, 0.95)	0.25	0.91	(0.86, 0.96)	0.96			
No	10	0.77	(0.68, 0.86)		0.74	(0.61, 0.87)				
*Index test bias*: Yes	12	0.89	(0.84, 0.95)	0.28	0.92	(0.85, 0.98)	0.17			
No	18	0.76	(0.69, 0.84)		0.89	(0.82, 0.95)				
*Reference test bias*: Yes	0									
No	16	0.83	(0.75, 0.89)		0.81	(0.71, 0.88)				
*Patient flow bias*: Yes	6	0.89	(0.79, 0.98)	0.37	0.89	(0.78, 1.00)	0.34			
No	27	0.83	(0.77, 0.89)		0.89	(0.83, 0.94)				
**TUBEX assay**	**12 (12)**	**0.75**	**(0.59, 0.85)**		**0.88**	**(0.84, 0.92)**		**22**	**(10, 47)**	**0.17**
*Patient selection bias*: Yes	6	0.77	(0.60, 0.94)	0.94	0.91	(0.86, 0.96)	0.01			
No	6	0.71	(0.51, 0.91)		0.86	(0.81, 0.92)				
*Index test bias*: Yes	3	0.55	(0.22, 0.88)	0.13	0.95	(0.91, 0.98)	0.07			
No	9	0.79	(0.67, 0.91)		0.85	(0.80, 0.89)				
*Reference test bias*: Yes	0									
No	12	0.75	(0.59, 0.85)		0.88	(0.84, 0.92)				
*Patient flow bias*: Yes	5	0.67	(0.44, 0.90)	0.24	0.9	(0.85, 0.96)	0.01			
No	7	0.79	(0.64, 0.93)		0.87	(0.82, 0.92)				
**Anti-*S*. *typhi* assay**	**13 (9)**	**0.75**	**(0.65, 0.82)**		**0.83**	**(0.76, 0.89)**		**15**	**(8, 27)**	**0**
*Patient selection bias*: Yes	7	0.79	(0.68, 0.89)	0.79	0.8	(0.70, 0.90)	0.02			
No	5	0.68	(0.53, 0.83)		0.86	(0.78, 0.95)				
*Index test bias*: Yes	3	0.89	(0.82, 0.97)	0.7	0.77	(0.61, 0.94)	0.05			
No	10	0.69	(0.61, 0.77)		0.85	(0.78, 0.92)				
*Reference test bias*: Yes	0									
No	6	0.67	(0.62, 0.71)		0.87	(0.79, 0.92)				
*Patient flow bias*: Yes	3	0.89	(0.82, 0.97)	0.7	0.77	(0.61, 0.94)	0.05			
No	10	0.69	(0.61, 0.77)		0.85	(0.78, 0.92)				
**Typhidot assay**	**20 (17)**	**0.84**	**(0.73, 0.92)**		**0.8**	**(0.67, 0.89)**		**22**	**(9, 57)**	**0.05**
*Patient selection bias*: Yes	9	0.84	(0.68, 0.99)	0.57	0.87	(0.75, 0.98)	0.96			
No	9	0.85	(0.71, 0.99)		0.79	(0.63, 0.95)				
*Index test bias*: Yes	2	0.5	(-0.02, 1.00)	0.14	0.79	(0.47, 1.00)	1			
No	13	0.84	(0.72, 0.95)		0.8	(0.68, 0.92)				
*Reference test bias*: Yes	0									
No	14	0.78	(0.62, 0.89)		0.85	(0.71, 0.93)				
*Patient flow bias*: Yes	5	0.63	(0.34, 0.91)	0.02	0.88	(0.74, 1.00)	0.58			
No	15	0.89	(0.81, 0.96)		0.77	(0.63, 0.91)				
**Widal assay (any antigen)**	**65 (51)**	**0.69**	**(0.61, 0.75)**		**0.83**	**(0.77, 0.88)**		**11**	**(7, 17)**	**0.05**
*Patient selection bias*:^1^ Yes	29									
No	31									
*Index test bias*: Yes	19	0.68	(0.57, 0.79)	0.24	0.88	(0.81, 0.95)	0.21			
No	34	0.65	(0.57, 0.74)		0.79	(0.71, 0.87)				
*Reference test bias*: Yes	0									
No	42	0.66	(0.57, 0.73)		0.84	(0.76, 0.90)				
*Patient flow bias*: Yes	13	0.73	(0.59, 0.87)	0.41	0.8	(0.67, 0.93)	0.04			
No	52	0.68	(0.60, 0.75)		0.84	(0.78, 0.90)				

Summary diagnostic accuracies of index tests with five or more comparisons and blood culture as the reference test. Meta-analysis performed using bivariate random effects binomial regression.

^1^ Could not be determined.

#### Index tests detecting antibodies

Antibody-based index tests were the most abundant, with 293 comparisons, of which 133 were Widal assays. The non-Widal assays ranged in publication year from 1979 to 2013. Analyte specificity varied and included the following antigen-specific antibody responses: flagellin (2), lipopolysaccharide (LPS, 43), LPS and flagellin (1), membrane preparation (3), outer membrane protein (OMP, 50), porins (1), whole *S*. *typhi* (20), unspecified Salmonella (2), O-9 analyte (17), Vi antigen (7), O and/or H antigen (10), O, H, and Vi antigen (1), and an unspecified analyte (3). Of the assays using the OMP analyte, 41 were the commercially available kit Typhidot (Reszon Diagnostics International, Malaysia). Additionally, all of the 17 assays using the O-9 analyte were the commercially available kit TUBEX^®^ *TF* (IDL, Sweden). Specimens used were most often serum, with two assays using lymphocyte culture supernatant, two using blood, one using plasma, and two using saliva. Immunoglobulin (Ig) isotypes evaluated included IgA (7), IgM (53), IgG (28), IgM and IgG together (30), and IgM, IgG, and IgA together (3); 39 studies did not specify the Ig isotype evaluated. Reference tests included PCR (3), Widal assays (10), culture (122), clinical features (1), and composite references (24).

Investigating only comparisons that used blood culture as a reference test, four index analytes were evaluated with five or more comparisons: anti-LPS, anti-*S*. *typhi*, TUBEX, and Typhidot. Anti-LPS assays compared to blood culture (n = 33) had the following summary results: Sens = 84% (78–89%), Spec = 89% (83–93%), DOR = 42 (23–75) (Table 2). TUBEX assays compared to blood culture (n = 12) had the following summary results: Sens = 75% (59–85%), Spec = 88% (84–92%), DOR = 22 (10–47) (Table 2). Anti-*S*. *typhi* assays compared to blood culture (n = 13) had the following summary results: Sens = 75% (65–82%), Spec = 83% (76–89%), DOR = 15 (8–27) (Table 2). Typhidot assays compared to blood culture (n = 20) had the following summary results: Sens = 84% (73–92%), Spec = 80% (67–89%), DOR = 22 (9–57). (Table 2)

The Widal assays ranged in publication year from 1977 to 2013. Among these assays, the analytes assessed included O antigen alone (58), H antigen alone (31), O and H antigens together (32), and unspecified antigen (12). Titer cutoffs of the Widal assays also varied by study, ranging from 1:20 to 1:640, with many unspecified (25). Reference tests included PCR (3), antibody-based assays (11), clinical features (11), culture (94), and composite references (14). Restricting analysis to comparisons with blood culture as the reference test, the Widal assay summary results were (n = 65): Sens = 69% (61–75%), Spec = 83% (77–88%), DOR = 11 (7–17). (Table 2)

**Fig 3:**
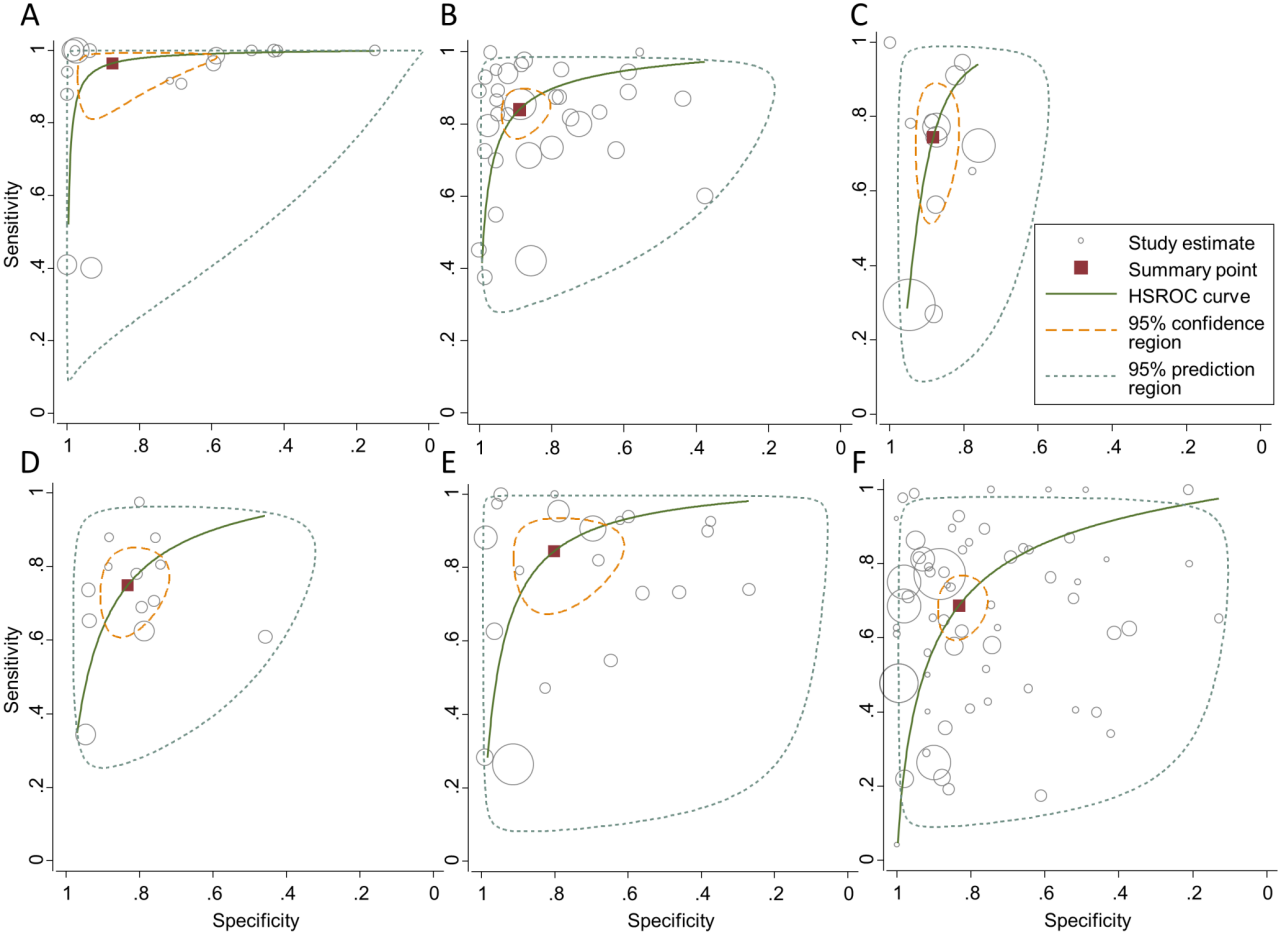
Meta-analysis results. Graphical illustration of sensitivities (y-axis) and specificities (x-axis) corresponding to comparisons included in the meta-analysis: PCR-based assays (A), anti-LPS assays (B), TUBEX^®^ assays (C), anti-*S*. *typhi* assays (D), Typhidot assays (E), Widal assays (F). Meta-analysis was performed using bivariate random effects binomial regression (STATA command: *metandi*). Sizes of individual study estimates (grey circle) represent sample size. Summary point (red square), hierarchical summary receiver operating characteristic curves (green line), 95% confidence regions (yellow dashed line), and 95% prediction regions (grey dashed line) are depicted.

A graphical illustration of sensitivities (y-axis) and specificities (x-axis) corresponding to the above comparisons included in the meta-analysis are presented in Fig 3, including the 95% confidence and prediction regions, and the hierarchical summary receiver operating characteristic curves. Additionally, forest plots of the meta-analysis results are provided in supplementary material (S2 Text). When assessing whether study quality had an effect on observed diagnostic accuracy, studies with patient selection bias had a significantly different specificity compared to studies without this bias for PCR, TUBEX, and anti-*S*. *typhi* assays, though the direction of the effect was not consistent across assays. Patient flow bias significantly affected specificity for TUBEX, anti-*S*. *typhi*, and Widal assays and sensitivity for Typhidot assays. Index test bias significantly affected sensitivity for PCR and specificity for anti-*S*. *typhi* assays. Finally, risk of reference test bias was unclear for many studies, based on the defined criteria, hampering interpretation of observed effect on diagnostic accuracy. (Table 2)

### Constructed numerical example

At a prevalence of 20% with a 50% sensitive and 100% specific test A, when the index test and test A are conditionally independent, the index test compared to test A provides an unbiased estimate of the true sensitivity of the index test, but underestimates specificity. When the index test is conditionally dependent on test A among subjects declared as “diseased” with a correlation of 0.4, the index test compared to test A appears to overestimate sensitivity considerably. Specificity is still underestimated, but to a lesser extent compared to the conditionally independent scenario. (Table 3)

**Table 3:** Constructed numerical example.

	All tests independent conditional on disease status		Index test conditionally dependent on test A among diseased (correlation = 0.4) and independent of test B		Index test conditionally dependent on test B among both diseased and non-diseased (correlation = 0.4) and independent of test A		Index test conditionally dependent on test B among both diseased and non-diseased (correlation = 0.7) and independent of test A	
	Sensitivity	Specificity	Sensitivity	Specificity	Sensitivity	Specificity	Sensitivity	Specificity
Index test compared to test A	80%	82.2%	96.0%	84.0%	NA	NA	NA	NA
Index test compared to test B	51.0%	87.0%	NA	NA	66.8%	93.5%	78.6%	98.3%
Index test compared to CRS	52.5%	85.2%	53.2%	85.4%	65.6%	89.4%	75.4%	92.6%

Assumed sensitivity and specificity of the three tests: index test, 80% and 90%; test A, 50% and 100%; test B, 85% and 85%. Comparing the index test to a CRS = (fever) AND ((test A positive) OR (test B positive)). Fever, test A, and test B are independent conditional on disease status. Index test is independent of fever conditional on disease status.

For test B with 85% sensitivity and 85% specificity, when the index test and test B are conditionally independent, the index test compared to test B underestimates sensitivity considerably. Specificity is only slightly underestimated. Note that the underestimation of sensitivity using test B as the reference results partially from the imperfect specificity of test B and the good sensitivity of the index test. A portion of subjects declared as diseased by test B are actually non-diseased and will tend to be declared as “non-diseased” by the index test, making the index test appear less sensitive than it actually is. Increasing the correlation between the index test and test B to 0.4 among diseased and non-diseased, the underestimation in sensitivity is decreased while specificity is slightly overestimated. When the correlation is increased to 0.7, the sensitivity estimate of the index test using test B as the reference is closer to the true sensitivity of the index test, with overestimation in specificity further increased.

When all tests are conditionally independent, results comparing the index test to the CRS appear similar to those comparing the index test to test B, with a slightly larger sensitivity estimate and smaller specificity estimate. When the index test is conditionally dependent on test A among diseased with a correlation of 0.4 and conditionally independent of test B, a slight increase in sensitivity and decrease in specificity are observed. If the index test is instead conditionally dependent on test B with a correlation of 0.4 among diseased and non-diseased and conditionally independent of test A, the index test compared to the CRS has an observed sensitivity closer to that using test B as the reference, with the observed specificity approximating true specificity. When the correlation is increased to 0.7, the index test compared to the CRS will slightly underestimate sensitivity and approximate the true specificity. This observed sensitivity and specificity are closest to truth among all comparisons, in the sense that the absolute bias is less than 5% for both sensitivity and specificity.

In S4 Table, we also explored results varying disease prevalence from 5% to 70%. Disease prevalence alters the interpretation of the observed performance of the index test relative to the reference test. There are some general patterns in the data: the specificity of the index test differs from truth with less magnitude for lower prevalence values, for most combinations of reference test and its correlation with index test explored; and, the sensitivity of the index test differs from truth with less magnitude at higher prevalence values, for most combinations of reference test and its correlation with index test explored. For some combinations of reference test and its correlation with index test, there are, however, switches in the directionality of the differences as prevalence changes (see S4 Table). Moreover, when disease prevalence is low (5%), using a reference test with imperfect sensitivity (50%) but perfect specificity gives an observed sensitivity and specificity fairly close to their true value when the index test is independent of the reference test conditional on disease status, not necessitating the use of a CRS. However, for larger prevalence, a reference test with low sensitivity can lead to severe bias in observed specificity, which may be alleviated with the use of a CRS. Interested readers can use the R code presented in S1 Text to explore the impact of parameter settings on the comparison between observed and true performance of the index test.

## Discussion

Based on our systematic review, numerous reference tests have been used to evaluate new typhoid diagnostics, with blood culture being the most commonly used test. Most studies included fever as an inclusion criterion, and further classified patients as having typhoid fever if fever reached a duration and/or temperature cutoff, which varied by study. A clinical criterion such as fever, in combination with another reference test, is a composite reference, though often it was not shown as such. Additionally, there were many studies that clearly stated their use of a composite reference, demonstrating willingness to use and general acceptance of composite reference standards by typhoid researchers. In the 21 studies that stated use of a CRS as the reference method, however, no two studies employed an identical CRS. The individual tests included in the CRS varied and were not well standardized, causing the resulting CRS to also vary and muddle comparisons across studies.

To evaluate the diagnostic accuracy of common index tests, a single reference test was used for comparison. Blood culture, the most common reference test used because of its perfect specificity, has unacceptably low sensitivity for many populations. Culturing more blood may improve the sensitivity of the test, as one setting demonstrated a sensitivity of 87.5% when using automated blood culture with 10 ml of blood [194]. Another study among children older than 60 days observed that the proportion of blood culture positives increased with each ml of blood cultured [195]. Though culturing 10 ml of blood may improve the diagnostic accuracy of blood culture, it may not be a feasible volume to obtain from patients, especially children, who are most impacted by the disease.

The meta-analysis provided a systematic process without necessitating assumptions to evaluate the accuracy of common index tests; however, the accuracy measures determined in the meta-analysis are still biased by the inaccuracy of the reference test. Antibody-based assays may be informative but standardization is essential. The available data are too varied in assay methods, Ig isotypes examined, and interpretation of results to form strong conclusions about a particular test’s diagnostic accuracy. More extensive evaluation of specific assays with few comparison studies may highlight promising tools. Additionally, there is the issue of regional variation in antibody responses. Healthy populations in regions with highly endemic typhoid fever have greater disease-specific antibody titers compared to regions with low or little typhoid fever, requiring diagnostic titer cutoffs to vary by region [7,194]. Based on our findings, PCR demonstrates promising results so far; however, fewer studies have been performed and these methods are still being modified and validated. Finally, study quality significantly impacted measures of sensitivity and specificity, though not to the same degree across assays. Statistical methods accounting for imperfect reference tests, such as latent class analysis, may help inform the selection of the CRS components, as well as its validation [9]. Latent class analysis offers additional benefits to dealing with an imperfect reference test compared to a CRS; however, the straightforward nature of calculating a CRS is preferable for routine practice.

In the constructed numerical example we considered combining blood culture, which has low sensitivity and perfect specificity, along with fever and another test with good sensitivity and specificity to generate a CRS that has improved sensitivity over blood culture at the cost of some specificity. Though actual performance of a CRS cannot be determined by this method, it explores how a CRS may compare to standalone reference tests in the evaluation of a new index test. We observed that the performance of the index test compared to the CRS depends on the correlation between the index test and the components of the CRS, which should be taken into account when interpreting the observed sensitivity and specificity of an index test relative to a CRS. Additionally, a CRS using an “or” statement will increase the reference test positives (true positives and false negatives) compared to using a single component of the CRS. A small increase in reference test positives will have a greater effect on observed sensitivity than specificity when the absolute number of reference test positives is much less than the number of reference test negatives, such as in low-prevalence studies. Additionally, high prevalence settings may benefit more from use of a CRS instead of a single imperfect test as reference.

The goal of this work is to start the conversation about the best components for a typhoid CRS and encourage agreement and adoption of a standardized composite reference for evaluating new typhoid diagnostics by all investigators. A standardized CRS will enable rigorous comparison of diagnostic accuracy data across studies, which is often difficult because of varying study designs and reference standards. We acknowledge the difficulties in standardizing a composite reference within the context of scarce resources. Strong advocacy by typhoid opinion leaders will be required to translate recommendations into consensus, and consensus into consistent action by the community. In order to validate the diagnostic accuracy of a new test, results from multiple studies in diverse regions will be needed. Additionally, use of a CRS would lead to improved confidence in prevalence estimates, which may help guide typhoid vaccination efforts. While a new CRS for typhoid may still be imperfect compared to diagnostic truth, there is much to gain from the adoption of a standardized composite reference.

In addition to the findings above, which are based on reviewing the literature, conducting a meta-analysis, and exploring a constructed numerical example, we think there are a number of considerations that are also important. First, one agreed upon composite reference should be adopted by all investigators to enable the leveraging of multiple studies in rigorous meta-analysis. That CRS should not include new technologies that are candidates for evaluation by the CRS, as this biases accuracy measures [10]. For example, newer technologies such as PCR may demonstrate superior diagnostic accuracy to the tests included in the CRS; however, studies evaluating these technologies are few, and their inclusion in the current CRS would prevent further validation and potential adoption in the future.

To achieve reproducibility, individual reference test components in the CRS need to be standardized. Clinical signs used should match the WHO definition of suspected typhoid fever: ≥37.5°C for ≥3 days. This feature may be considered a screening criteria or a component of the CRS using an AND rule (fever positive AND positive based on some algorithm of other tests), depending on study design. The important issue is that CRS positive cases also meet the WHO definition for suspected typhoid fever. Also, if blood culture is used, it is necessary to specify blood volume and media or culture system, at a minimum. Including blood culture is recommended because of its widespread use, 100% specificity, and ability to isolate the organism for further investigation, such as drug resistance. Additionally, a commercially available antibody-based assay, which is technologically feasible in many settings, is more likely to generate reproducible results through use of a standardized product and protocol, though issues with performance variability can still arise. Only two commercially available antibody-based assays had been evaluated in a sufficient number of studies to be summarized in our meta-analysis, TUBEX *TF* and Typhidot. In a recent meta-analysis comparing the same two tests, the TUBEX assay showed comparable summary sensitivity and specificity as this study (69% and 88%, respectively), while sufficient studies were not available to evaluate Typhidot by the same metrics [196].Of note: the current manufacturer of TyphiDot (Reszon Diagnostics International Sdn. Bhd., Selangor, Malaysia) has only been making this test since ca. 2010, and many of the studies that established the diagnostic accuracy of this product were performed with the tests made by the previous manufacturer (Malaysian Bio-Diagnostic Research, Sdn. Bhd, Bangi, Malaysia). To our knowledge, there is no published equivalence data that establishes continuity in performance after the change in manufacturing site, which may warrant additional evaluation studies.

Finally, studies should use a prospective cohort design and clearly specify inclusion and exclusion criteria. Patient selection bias significantly impacted the observed specificity of several evaluated index tests (PCR, TUBEX, anti-*S*. *typhi* assays). Covariates such as age, fever duration, and prior antibiotic use may be important to consider, though there were insufficient data to evaluate these variables in this study. Ultimately, the proposed CRS should be viewed as a dynamic diagnostic tool. Evaluation and revision as newer technologies are validated and demonstrate improved diagnostic accuracy is encouraged, with the ultimate goal of identifying a robust gold standard.

Strengths of this study include its thorough review of all available published literature evaluating two diagnostic tests for the detection of typhoid fever. In addition, rigorous statistical techniques were utilized to summarize the diagnostic accuracy of similar comparisons across studies. Limitations in this work arose from the diversity of diagnostic tests used by different investigators, and how those tests were performed. As a result, many comparisons could not be included in the meta-analysis or had to be summarized in broader groups. Incomplete description of methods and confounding factors also limited our ability to assess heterogeneity across studies. Finally, this analysis was based on published literature only; however, publication bias was not detected in the meta-analysis, as assessed by Deeks’ funnel plot asymmetry test (p>0.05) [197].

Better diagnostic tests to detect typhoid fever are needed to improve disease burden estimates and potentially accelerate the adoption of new typhoid vaccines where they are needed most [198,199]. For this to happen, standardization, consensus, and broad adoption of a single gold standard based on a composite reference are required so that new technologies can be confidently judged for their diagnostic accuracy. Additionally, uniform reporting of diagnostic test accuracy study results conforming to the STARD guidelines and QUADAS-2 tool should remain the standard to enable facile comparison of studies [182,200]. Necessary next steps include a broad discussion by stakeholders of the merits of a CRS to build consensus, and selection of the CRS components, followed by validation of the CRS in a well-designed clinical study. The work presented in this paper is an important initial step, and combined with statistical analyses such as latent class analysis, may further increase confidence in using a CRS. Ultimately, using a CRS as an improved gold standard for typhoid fever will contribute to an increased awareness of the real global cost of the typhoid epidemic.

## Supporting Information

S1 Table PRISMA Checklist. (DOC)

S2 Table Systematic review search terms. (DOCX)

S3 Table Index/reference combinations. (XLSX)

S4 Table Constructed numerical example: Difference from true accuracy at 5%, 20%, and 70% prevalence. (DOCX)

S1 Text Detailed derivations for constructed numerical example. (DOCX)

S2 Text Forest plots of meta-analysis results. (DOCX)
