# Supplementary material for: A Meta-Analysis of Typhoid Diagnostic Accuracy Studies: A Recommendation to Adopt a Standardized Composite Reference
Source: PLoS One. 2015 Nov 13;10(11):e0142364. doi: 10.1371/journal.pone.0142364 (PMC4643909; doi:10.1371/journal.pone.0142364)
Supplement: S2 Table — (DOCX) [file pone.0142364.s002.docx]

**S2 Table. Search Terms**

1. Database: PubMed Limits on all searches: human; English language

Exclusions (terms): non-typhoid salmonella; food-borne illness; diarrheal diseases; gastroenteritis; Salmonella enteritidis; Salmonella typhimurium; vector-borne diseases; malaria; dengue

Exclusions (publication types): commentary; editorial; non-comparison studies

| **general concept** | **search terms - MeSH** | **search terms - text words** |  |
| --- | --- | --- | --- |
| **pathogens** |  |  |  |
| salmonella | Salmonella; Salmonella enterica; Salmonella typhi; Salmonella paratyphi A; Salmonella paratyphi B; Salmonella paratyphi C; salmonella infections | typhoid; typhoid fever; S.typhi; S.paratyphi A; S.paratyphi B; S.paratyphi C; salmonella serovar |  |
| **conditions** |  |  | |
| febrile illness | fever | febrile illness; pyrexia | |
| **diagnostics** |  |  | |
| diagnosis | diagnosis | diagnosis | |
| diagnostic tests | diagnostic tests, routine; diagnostic techniques; reagent kits, diagnostic | diagnostic tests | |
| specificity/specificity | sensitivity and specificity | Sensitivity; specificity | |
| accuracy | predictive value of tests | accuracy | |
| bacteria detection | bacteria/isolation & purification; bacteriological techniques | bacteriological tests | |
| microbiology | colony count, microbial; microbial sensitivity tests; molecular diagnostic techniques | microbiology | |
| lab tests | laboratory techniques and procedures; clinical laboratory techniques | lab test; laboratory test | |
| urine tests | urine/microbiology; urinalysis | urine test | |
| blood tests | hematological tests; serologic tests | blood test; Widal test; Tubex test; Typhidot test | |
| polymerase chain reaction | polymerase chain reaction ; reverse transcriptase polymerase chain reaction | PCR; RT-PCR | |
| immunoassay | Immunoassay; immunoenzyme techniques; enzyme-linked immunosorbent assay; enzyme multiplied Immunoassay technique; immunologic tests | ELISA | |
| latex agglutination test | latex fixation tests; agglutination tests | latex agglutination test | |
| gene amplification | genetic techniques; nucleic acid amplification techniques; DNA, bacterial; nucleic acids; RNA, bacterial; genes, bacterial; gene amplification | gene amplification | |
| cell culture | cell culture techniques; culture techniques | cell culture; lab culture; laboratory culture | |
| microscopy | microscopy | microscopy | |
| composite reference method | reference standards | composite reference; composite reference method; composite reference standard; imperfect reference | |

1. Database: EMBASE Limits on all searches: humans; English language

Exclusions: non-typhoid salmonella; food-borne illness; diarrheal diseases; gastroenteritis; Salmonella enteritidis; Salmonella typhimurium; vector-borne diseases; malaria; dengue

Exclusions (publication types): commentary; editorial; non-comparison studies

| **general concept** | **search terms - EMTREE** | **search terms - text words** |  |
| --- | --- | --- | --- |
| **pathogen** |  |  |  |
| salmonella | salmonella | salmonella infections; Salmonella enterica; Salmonella typhi; Salmonella paratyphi A; Salmonella paratyphi B; Salmonella paratyphi C; typhoid; typhoid fever; S.typhi; S.paratyphi A; S.paratyphi B; S.paratyphi C; salmonella serovar |  |
| **conditions** |  |  |  |
| febrile illness | fever | fever; pyrexia |  |
| **diagnostics** |  |  | |
| diagnosis | diagnosis |  | |
| diagnostic tests | diagnostic test; diagnostic procedure | diagnostic tests | |
| specificity/specificity | sensitivity and specificity |  | |
| accuracy | diagnostic value | accuracy | |
| bacteria detection | bacterial diagnosis; bacterium detection; bacterium culture | bacteriological techniques | |
| microbiology | microbiology; microbiological assay | microbiology | |
| lab tests | laboratory diagnosis; laboratory test | lab test; laboratory tests | |
| urine tests | urinalysis | urine tests | |
| blood tests | serodiagnosis; blood analysis | blood test; serologic tests; Widal test; Tubex test; Typhidot test | |
| polymerase chain reaction | polymerase chain reaction; reverse transcriptase polymerase chain reaction | PCR; RT-PCR | |
| immunoassay | Immunoassay | ELISA; enzyme-linked immunosorbent assay; enzyme multiplied immunoassay technique; immunologic tests | |
| latex agglutination test | latex agglutination test | latex agglutination test | |
| gene amplification |  | gene amplification; RNA; DNA | |
| cell culture | cell culture; cytodiagnosis; culture technique; | cell culture techniques | |
| microscopy | microscopy | microscopy | |

1. Database: ISI Web of Science Limits on all searches: humans; English language

Exclusions: non-typhoid salmonella; food-borne illness; diarrheal diseases; gastroenteritis; Salmonella enteritidis; Salmonella typhimurium; vector-borne diseases; malaria; dengue

Exclusions (publication types): commentary; editorial; non-comparison studies

| **general concept** | **controlled vocabulary not available** | **search terms - TOPIC** | |
| --- | --- | --- | --- |
| **pathogen** |  |  | |
| salmonella |  | salmonella ; salmonella infections; Salmonella enterica; Salmonella typhi; Salmonella paratyphi A; Salmonella paratyphi B; Salmonella paratyphi C; typhoid; typhoid fever; S.typhi; S.paratyphi A; S.paratyphi B; S.paratyphi C; salmonella serovar | |
| **conditions** |  |  | |
| febrile illness |  | febrile illness; fever; pyrexia | |
| **diagnostics** |  |  |  |
| diagnosis |  | diagnosis |  |
| diagnostic tests |  | diagnostic tests; diagnostic procedure; diagnostic technique |  |
| specificity/specificity |  | sensitivity and specificity |  |
| accuracy |  | accuracy |  |
| bacteria detection |  | bacteriological culture; bacteria culture |  |
| microbiology |  | microbiology |  |
| lab tests |  | laboratory diagnosis; laboratory test ; lab test; laboratory tests |  |
| urine tests |  | urine tests; urinalysis |  |
| blood tests |  | blood test; serologic tests; serodiagnosis; Widal test; Tubex test; Typhidot test |  |
| polymerase chain reaction |  | polymerase chain reaction; reverse transcriptase polymerase chain reaction ; PCR; RT-PCR |  |
| immunoassay |  | immunoassay ; ELISA; enzyme-linked immunosorbent assay; immunologic tests |  |
| latex agglutination test |  | latex agglutination test |  |
| gene amplification |  | gene amplification; RNA; DNA |  |
| cell culture |  | cell culture techniques; cell culture |  |
| microscopy |  | microscopy |  |
