# Supplementary material for: A Meta-Analysis of Typhoid Diagnostic Accuracy Studies: A Recommendation to Adopt a Standardized Composite Reference
Source: PLoS One. 2015 Nov 13;10(11):e0142364. doi: 10.1371/journal.pone.0142364 (PMC4643909; doi:10.1371/journal.pone.0142364)
Supplement: S4 Table — (DOCX) [file pone.0142364.s004.docx]

**S4 Table. Constructed numerical example: Difference from true accuracy at 5%, 20%, and 70% prevalence.**

|  | **All tests independent conditional on disease status** | | | | | | **Index test conditionally dependent on test A among diseased (correlation = 0.4) and independent of test B** | | | | | | **Index test conditionally dependent on test B among both diseased and non-diseased (correlation = 0.4) and independent of test A** | | | | | | **Index test conditionally dependent on test B among both diseased and non-diseased (correlation = 0.7) and independent of test A** | | | | | |
| --- | --- | --- | --- | --- | --- | --- | --- | --- | --- | --- | --- | --- | --- | --- | --- | --- | --- | --- | --- | --- | --- | --- | --- | --- |
|  | **Difference From True Sensitivity (%)** | | | **Difference From True Specificity (%)** | | | **Difference From True Sensitivity (%)** | | | **Difference From True Specificity (%)** | | | **Difference From True Sensitivity (%)** | | | **Difference From True Specificity (%)** | | | **Difference From True Sensitivity (%)** | | | **Difference From True Specificity (%)** | | |
| **Prevalence (%)** | **5** | **20** | **70** | **5** | **20** | **70** | **5** | **20** | **70** | **5** | **20** | **70** | **5** | **20** | **70** | **5** | **20** | **70** | **5** | **20** | **70** | **5** | **20** | **70** |
| Index test compared to test A | 0 | **0** | 0 | -2 | **-8** | -38 | +18 | **+18** | +18 | -1 | **-6** | -29 | 0 | **0** | 0 | -2 | **-8** | -38 | 0 | **0** | 0 | -2 | **-8** | -38 |
| Index test compared to test B | -54 | **-29** | -5 | -1 | **-3** | -20 | -54 | **-29** | -5 | -1 | **-3** | -20 | -30 | **-13** | +3 | +5 | **+4** | -6 | -13 | **-1** | +10 | +9 | **+8** | +5 |
| Index test compared to CRS | -53 | **-27** | -4 | -1 | **-5** | -29 | -52 | **-27** | -3 | -1 | **-5** | -27 | -30 | **-14** | 0 | +3 | **-1** | -23 | -14 | **-5** | +4 | +6 | **+3** | -18 |

Assumed sensitivity and specificity of the three tests: index test, 80% and 90%; test A, 50% and 100%; test B, 85% and 85%. Comparing the index test to a CRS = (fever) AND ((test A positive) OR (test B positive)). Prevalence assumed at 3 levels: 5%, 20%, and 70%. Fever, test A, and test B are independent conditional on disease status. Index test is independent of fever conditional on disease status.
