# Supplementary material for: A Meta-Analysis of Typhoid Diagnostic Accuracy Studies: A Recommendation to Adopt a Standardized Composite Reference
Source: PLoS One. 2015 Nov 13;10(11):e0142364. doi: 10.1371/journal.pone.0142364 (PMC4643909; doi:10.1371/journal.pone.0142364)
Supplement: S1 Text — (DOCX) [file pone.0142364.s005.docx]

**S1 Text. Detailed derivations for constructed numerical example**

**Notation:**

| Test Name | True Sens. | True Spec. |
| --- | --- | --- |
| Index Test: I={0,1} | P_I_ | q_I_ |
| Test A: A={0,1} | P_A_ | q_A_ |
| Test B: B={0,1} | P_B_ | q_B_ |
| Test F (fever): F={0,1} | P_F_ | q_F_ |

We use 0,1 to indicate whether a test is negative or positive.

CRS: (A | B) & F = C

Assumptions:

A┴B, A┴F, B┴F, I┴F conditional on D, where D=1, 0 denote diseased and non-diseased

1. **Derivation of sensitivity and specificity of CRS**

Sensitivity of CRS

P(C=1 | D=1) = P_c_ = P_r_ [ (A=1 or B=1) & F=1 | D=1]

= P_r_ (F=1 | D=1) - P_r_ (A=0 & B=0 & F=1 | D=1)

= P_F_ – (1 – P_A_) (1-P_B_) P_F_

Specificity of CRS

P(C=0 | D=0) = q_c_ = P_r_ [ (A=0 and B=0) or F=0 | D=0]

= 1 – P_r_ [ (A=1 or B=1) & F=1 | D=0]

= 1 – [P_r_(F=1 | D=0) – P_r_ (A=0 & B=0 & F=1 | D=0)]

= 1 – (1-q_F_) + q_A_q_B_ (1-q_F_)

= q_F_ + q_A_q_B_ x (1- q_F_)

1. **Derivation of the observed sensitivity and specificity of the index test using test A as the reference**

Suppose correlation between I and A conditional on D=1 is P_1_,

correlation between I and A conditional on D=0 is P_0_

Then

P_r_(I=1 and A=1 | D=1)

= E(IA | D=1) = E(I | D=1) x E(A | D=1) + P_1_ x [Var( I | D=1) x Var(A|D=0)]^1/2^

= P(I=1 | D=1) x P(A=1 |D=1 + P_1_ x [Var(I | D=1) x Var(A | D=1)]^1/2^ ,

where

Var(I | D=1) = P(I=1 | D=1) x P(I=0 | D=1)

Var(A | D=1) = P(A=1 | D=1) x P(A=0 |D =1 ).

And,

P_r_(I=1 and A=0 | D=1)

= P_r_ (A=0 | D=1) - P_r_ (I=1 and A=1 | D=1) ,

P_r_ (I=0 and A=0 | D=1)

= 1 - P_r_ (I=1 and A=1 | D=1) - P_r_(I=1 and A=0 | D=1)

- P_r_ (I=0 and A=1 | D=1).

Similarly,

P(I=1 and A=1 | D=0)

=E( IA | D=0)

=E(I | D=0) x E(A | D=0) + P_0_ x [var(I | D=0) x var(A | D=0)]^1/2^,

where var(I | D=0) = P(I=1 | D=0) x P(I=0 | D=0)

var(A|D =0)=P(A=1| D=0) x P(A=0 | D=0).

And,

P_r_(I=1 and A=0 | D=0)

=P_r_(A=0|D=0) – P_r_(I=1 and A=1 | D=0),

P_r_(I=0 and A=1 | D=0)

=P_r_(I=0 | D=0) – P_r_(I=1 and A=1 | D=0),

P_r_(I=0 and A=0 | D=0)

=1- P_r_(I=1 and A=1 | D=0) – P_r_(I=1 and A=0 | D=0)

- P_r_(I=0 and A=1 | D=0).

Finally, sensitivity of index test using test A as reference is

P(I=1 | A=1)

= (P(I=1 and A=1))/ (P(A=1))

=(P(I=1 and A=1 | D=0) x P(D=0) + P(I=1 and A=1 | D=1) x P(D=1)) / (P(A=1 | D=0) x P(D=0) + P(A=1| D=1) x P(D=1)).

Specificity of index test using test A as reference is

P(I=0|A=0)

=(P(I=0 and A=0)) / (P(A=0))

=(P(I=0 and A=0 | D=0) x P(D=0) + P(I=0 and A=0 | D=1) x P(D=1)) / (P(A=0 | D=0) x P(D=0) + P(A=0 | D=1) x P(D=1)).

1. **Derivation of sensitivity and specificity of the index test using CRS as reference**

Sensitivity of index test using CRS as reference is

P(I=1 | C=1)

=(P(I=1 and C=1)) / (P(C=1))

=(P(I=1 and (A=1 or B=1) and F=1 | D=1) x P(D=1)) + (P(I=1 and (A=1 or B=1) and F=1 | D=1) x P(D=0) / P(C=1 | D=1) x P(D=1) + P(C=1 | D=0) x P(D=0).

Note:

P(I=1 and (A=1 or B=1) and F=1 | D=1)

=P(I=1 and (A=1 or B=1) and F=1 | D=1) - P(I=1 and F=1 and A=0 and B=0 | D=1)

=P(I=1|D=1) x P(F=1 | D=1)

- P(I=1 and A=0 | D=1) x P(B=0 | D=1) x P(F=1 | D=1).

And,

P(I=1 and (A=1 or B=1) and F=1 | D=0)

=P(I=1 | D=0) x P(F=1 | D=0) - P(I=1 and A=0 | D=0) x P(B=0 | D=0) x P(F=1 | D=0).

Specificity of index test using CRS as reference is

P(I=0) | C=0)

=P(I=0) and C=0) / P(C=0)

=(P(I=0 and C=0 | D=1) x P(D=1) + P(I=0 and C=0 |D=0) x P(D=0)) / (P(C=0 | D=1) x P(D=1) + P(C=0 |D=0) x P(D=0).

Note,

P(I=0 and C=0 | D=1)

=P(I=0 |D=0) – P(I=0 and C=1 |D=1)

=P(I=0 |D=1) – P(I=0 and (A=1 or B=1) and F=1 | D=1)

=P(1=0 | D=1) – [P(I=0 | D=1) x P(F=1 | D=1) – P (I=0 and A=0 | D=1) x P(B=0 | D=1) x P(F=1 |D=1)].

And,

P(I=0 and C=0 | D=0)

=P(I=0 | D=0) – P(I=0 and C=1 | D=0)

=P(I=0 | D=0) – [P(I=0 |D=0) x P(F=1 | D=0) – P(I=0 and A=0 | D=0) x P(B=0 | D=0) x P(F=1 | D=0)].

**Example R code for computing observed sensitivity and specificity of index test relative to individual reference test or CRS**

sd.bin<-function(p){

sqrt(p*(1-p))

}

######################################################################

### Assumptions:

### Fever independent of test A and independent of test B

### Test A and test B independent conditional on disease status

### Index test independent of test B conditional on disease status

rho<-0.2 ### disease prevalence

pa<-0.5 ### true sensitivity of reference test A

qa<-1 ### true specificity of reference test A

pb<-0.85 ### true sensitivity of reference test B

qb<-0.85 ### true specificity of reference test B

pf<-0.8 ### true sensitivity of fever

qf<-0.2 ### true specificity of fever

pi<-0.8 ### true sensitivity of the index test

qi<-0.9 ### true specificity of the index test

##################

cor.ai.D<-0.4 ### correlation between index test and reference test A among diseased

cor.ai.Dbar<-0 ### correlation between index test and reference test A among non-diseased

####joint distribution of index test and test A conditional on disease status

(p.a1i1.D<-pa*pi+sd.bin(pa)*sd.bin(pi)*cor.ai.D)

(p.a1i0.D<-pa*(1-pi)-sd.bin(pa)*sd.bin(pi)*cor.ai.D)

(p.a0i1.D<-(1-pa)*pi-sd.bin(pa)*sd.bin(pi)*cor.ai.D)

(p.a0i0.D<-(1-pa)*(1-pi)+sd.bin(pa)*sd.bin(pi)*cor.ai.D)

(p.a1i1.Dbar<-(1-qa)*(1-qi)+sd.bin(qa)*sd.bin(qi)*cor.ai.Dbar)

(p.a1i0.Dbar<-(1-qa)*qi-sd.bin(qa)*sd.bin(qi)*cor.ai.Dbar)

(p.a0i1.Dbar<-qa*(1-qi)-sd.bin(qa)*sd.bin(qi)*cor.ai.Dbar)

(p.a0i0.Dbar<-qa*qi+sd.bin(qa)*sd.bin(qi)*cor.ai.Dbar)

####joint distribution of index test and CRS conditional on disease status

p.c1i1.D=pi*pf-p.a0i1.D*(1-pb)*pf

p.c1i1.Dbar=(1-qi)*(1-qf)-p.a0i1.Dbar*qb*(1-qf)

p.c0i0.D=(1-pi)-((1-pi)*pf-p.a0i0.D*(1-pb)*pf)

p.c0i0.Dbar=qi-(qi*(1-qf)-p.a0i0.Dbar*qb*(1-qf))

##################

### CRS is constructed as (fever) AND ((test A positive) OR (test B positive))

###

### sensitivity for CRS

(pc=pf-(1-pa)*(1-pb)*pf)

### specificity for CRS

(qc=qf+qa*qb*(1-qf))

#########################

##### observed sensitivity of index test relative to reference test A

(SEN.ia<-(p.a1i1.Dbar*(1-rho)+p.a1i1.D*rho)/((1-qa)*(1-rho)+pa*rho))

##### observed specificity of index test relative to reference test A

(SPE.ia<-(p.a0i0.Dbar*(1-rho)+p.a0i0.D*rho)/(qa*(1-rho)+(1-pa)*rho))

###################################

#### observed sensitivity of index test relative to CRS

(SEN.ic<-(p.c1i1.Dbar*(1-rho)+p.c1i1.D*rho)/((1-qc)*(1-rho)+pc*rho))

#### observed specificity of index test relative to CRS

(SPE.ic<-(p.c0i0.Dbar*(1-rho)+p.c0i0.D*rho)/(qc*(1-rho)+(1-pc)*rho))
