# Supplementary material for: A Meta-Analysis of Typhoid Diagnostic Accuracy Studies: A Recommendation to Adopt a Standardized Composite Reference
Source: PLoS One. 2015 Nov 13;10(11):e0142364. doi: 10.1371/journal.pone.0142364 (PMC4643909; doi:10.1371/journal.pone.0142364)

S**2 Text.** **Forest plots of meta-analysis results.** Graphical illustration of sensitivities (left), specificities (right), and confidence intervals corresponding to comparisons included in the meta-analysis: PCR-based assays (A), anti-LPS assays (B), TUBEX® assays (C), anti-*S. typhi* assays (D), Typhidot assays (E), and Widal assays (F). Meta-analysis was performed using bivariate random effects binomial regression.


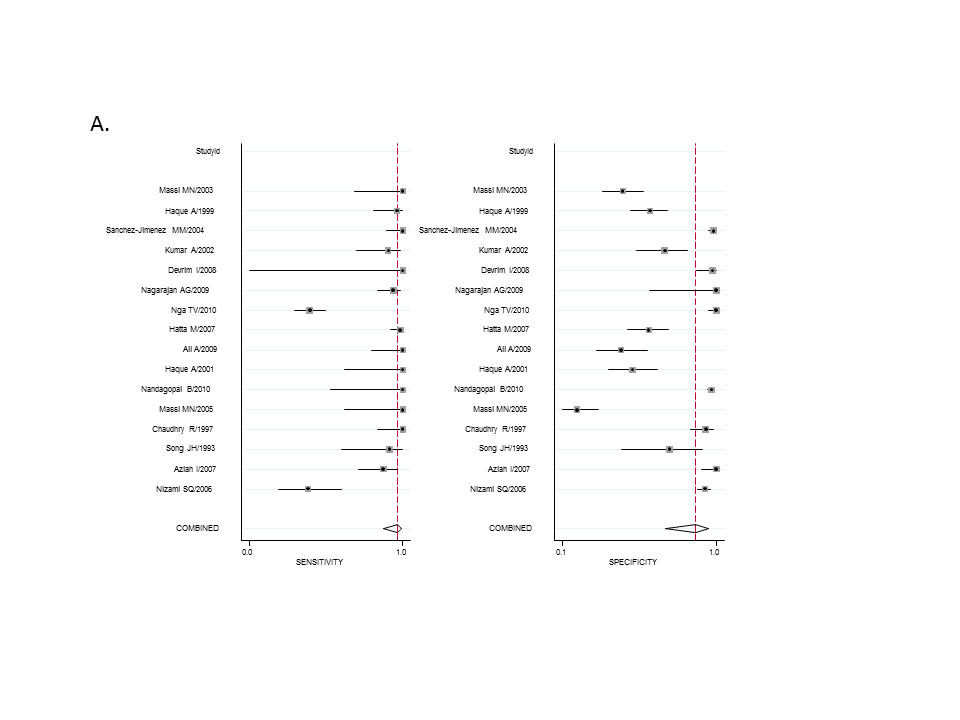


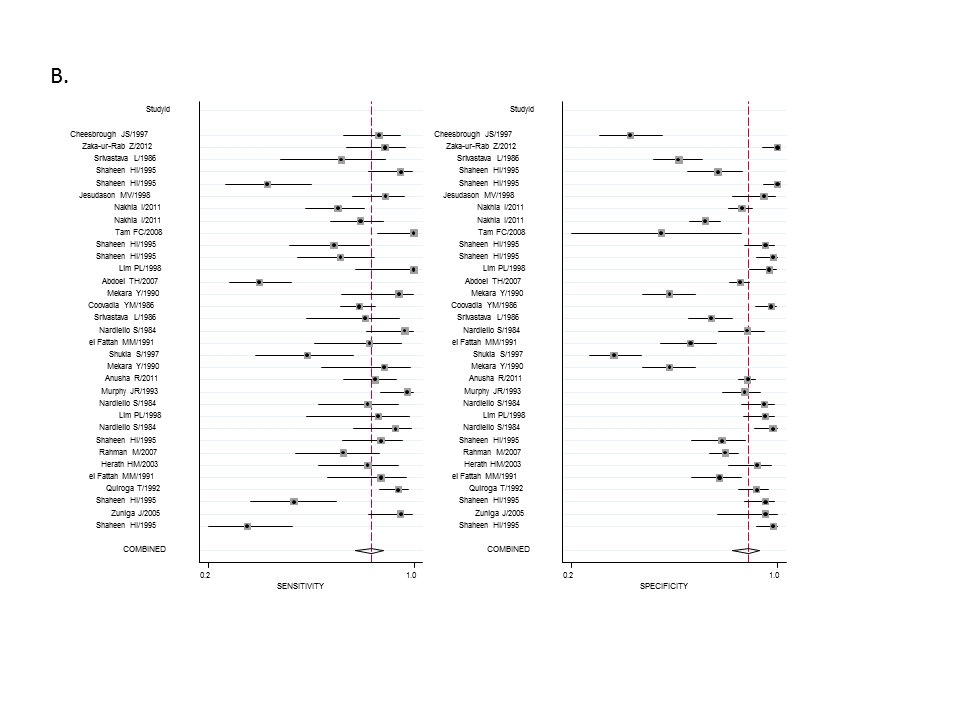


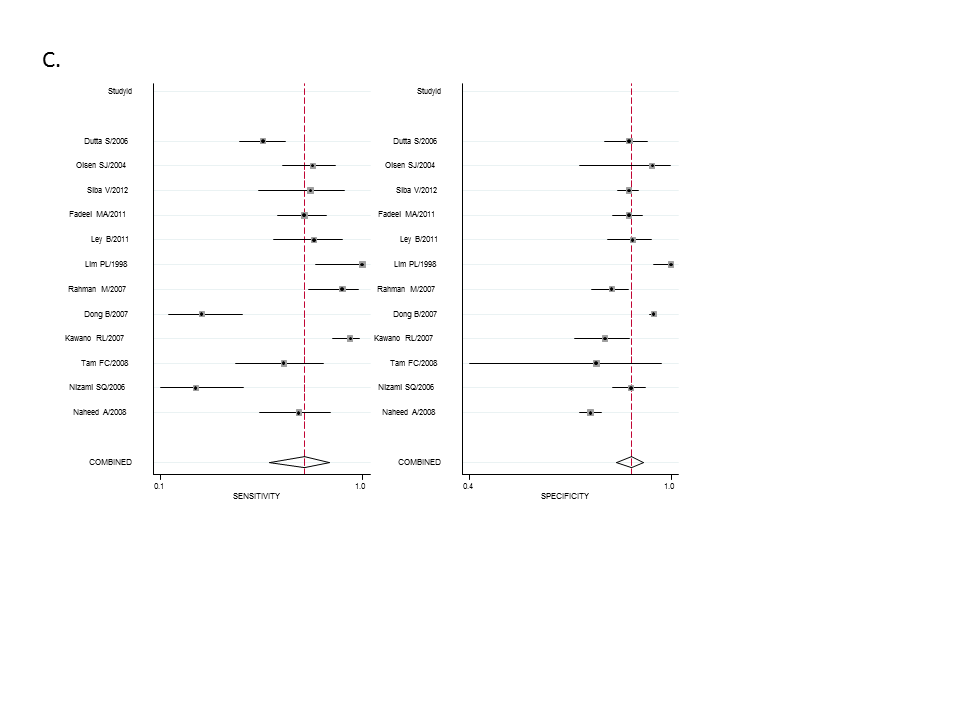

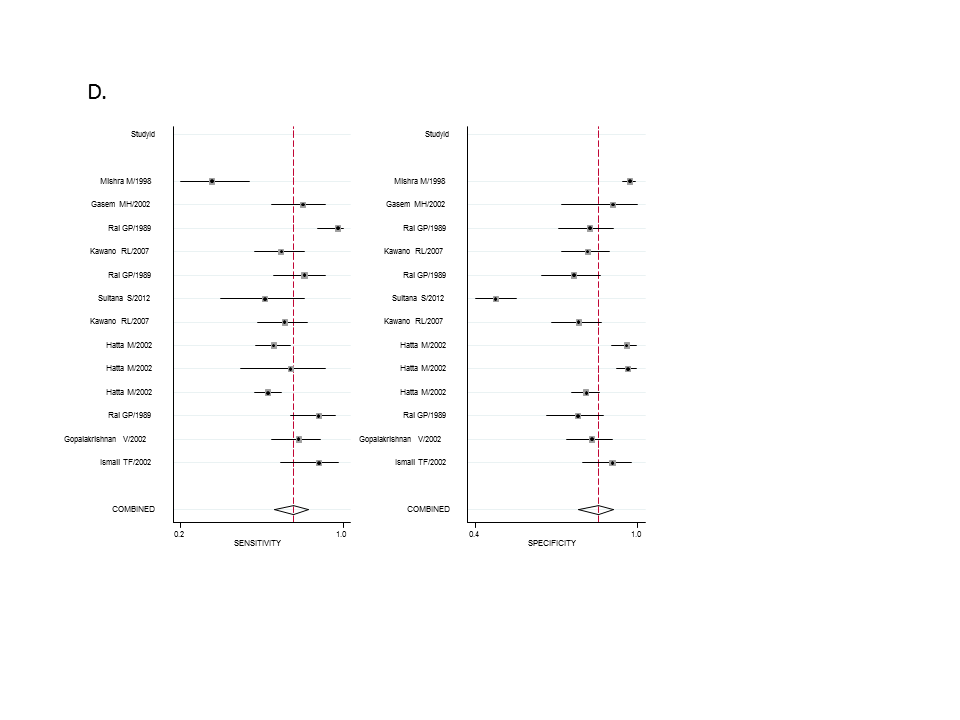

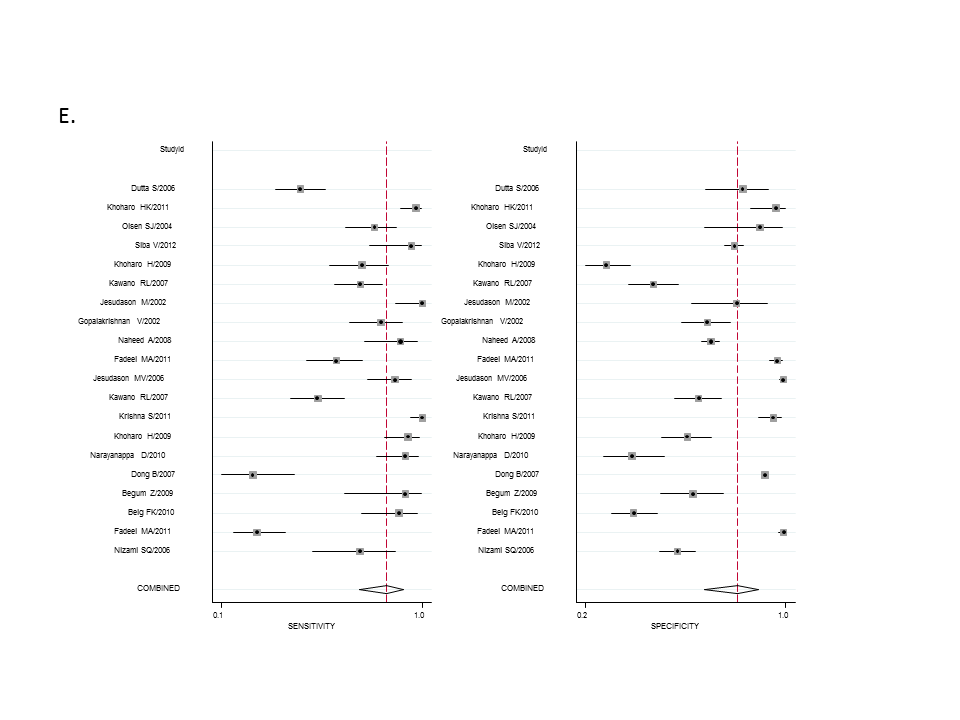

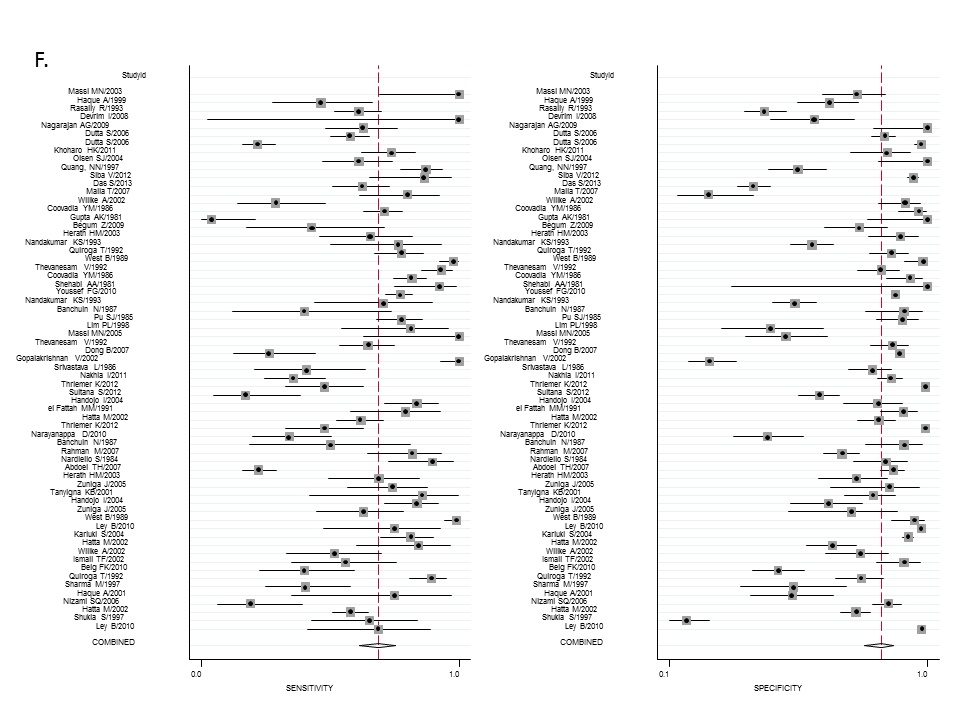

Supplement: S2 Text — (DOCX) [file pone.0142364.s006.docx]
